# Syntenin-1 promotes colorectal cancer stem cell expansion and chemoresistance by regulating prostaglandin E2 receptor

**DOI:** 10.1038/s41416-020-0965-9

**Published:** 2020-06-29

**Authors:** Kazuya Iwamoto, Hidekazu Takahashi, Daisuke Okuzaki, Hideo Osawa, Takayuki Ogino, Norikatsu Miyoshi, Mamoru Uemura, Chu Matsuda, Hirofumi Yamamoto, Tsunekazu Mizushima, Masaki Mori, Yuichiro Doki, Hidetoshi Eguchi

**Affiliations:** 1 Department of Gastroenterological Surgery, Osaka University Graduate School of Medicine, Osaka, Japan; 2 Department of Molecular Genetics, Research Institute for Microbial Diseases, Osaka University, Osaka, Japan; 3 Department of Surgery and Science, Kyushu University Graduate School of Medical Sciences, Fukuoka, Japan

**Keywords:** Cancer stem cells, Colorectal cancer, Tumour biomarkers, Chronic inflammation

## Abstract

**Background:**

The protein syntenin-1 is expressed by a variety of cell types, and is upregulated in various malignancies, including melanoma, breast cancer and glioma. Although the mechanism by which elevated syntenin-1 expression contributes to cancer has been described, the exact pathway has not been elucidated.

**Methods:**

To investigate the involvement of syntenin-1 in colorectal cancer (CRC), we performed immunohistochemical analysis of 139 CRC surgical specimens. We also examined syntenin-1 knockdown in CRC cell lines.

**Results:**

High syntenin-1 expression was associated with less differentiated histologic grade and poor prognosis, and was an independent prognostic indicator in CRC. Syntenin-1 knockdown in CRC cells reduced the presence of cancer stem cells (CSCs), oxaliplatin chemoresistance and migration. DNA microarray analysis and quantitative real-time polymerase chain reaction showed decreased prostaglandin E2 receptor 2 (PTGER2) expression in syntenin-1-knockdown cells. PTGER2 knockdown in CRC cells yielded the same phenotype as syntenin-1 knockdown. Celecoxib, which has anti-inflammatory effects by targeting cyclooxygenase-2, reduced CSCs and decreased chemoresistance, while prostaglandin E2 (PGE2) had the opposite effect.

**Conclusions:**

Our findings suggested that syntenin-1 enhanced CSC expansion, oxaliplatin chemoresistance and migration capability through regulation of PTGER2 expression. Syntenin-1 may be a promising new prognostic factor and target for anti-cancer therapies.

## Background

Despite the availability of advanced chemotherapeutic treatments, colorectal carcinoma (CRC) remains a major cause of cancer morbidity and mortality, representing the third most common type of cancer in men and the second most common in women worldwide.^1,2^ Several biomarkers have been investigated for relevance to CRC prognosis, including RAS, BRAF mutations and MSI status. Additionally, for some of these CRC phenotypes, effective treatments have emerged, such as MEK inhibitors and immune checkpoint inhibitors. In a recent report, colorectal cancer was classified according to consensus molecular subtype, and patients with CMS4 tumours showed worse overall survival and relapse-free survival.^3^ There is presently no effective treatment for CMS4 CRC.

Epidemiologic and experimental evidence strongly implicate chronic inflammation as a risk factor for the development of many cancers, including colorectal cancer. Most human colorectal cancers exhibit marked elevation of cyclooxygenase-2 (COX-2) expression.^4,5^ Moreover, the COX-2 and prostaglandin E2 (PGE2) receptor subtypes are reportedly involved in intestinal carcinogenesis and activation of multiple signalling pathways.^6–8^ PGE2 receptor 2 (PTGER2) and PGE2 receptor 4 (PTGER4) receptors activate PGE2, triggering a series of events,^9^ with several studies indicating that PGE2 mediates the effects of chronic inflammation on colorectal cancer stem cell (CSC) expansion and chemoresistance.^10,11^

Melanoma differentiation-associated gene-9, also known as syntenin-1, is a multifunctional scaffold protein that cross-talks with different classes of proteins and activates defined cell signalling pathways, thus regulating diverse physiologic and pathologic processes, including tumour progression and metastasis.^12,13^ Syntenin-1 reportedly links activated leucocyte cell adhesion molecule to the actin cytoskeleton in the dendritic cells, which play a key role in the immunoregulation.^14^ Syntenin-1 also plays critical roles in regulating various other forms of immunomodulation.^15–22^

Here we investigated whether syntenin-1 controls chronic inflammation signalling via PGE2, causing CSC cells to proliferate and acquire chemoresistance.

## Methods

### Patients and clinical samples

Tumour tissue and adjacent normal tissues were collected from CRC patients who underwent surgery between 2003 and 2006 at Osaka University Hospital. Fresh tumour tissue specimens were cut from the tumours, and corresponding adjacent normal tissue specimens were taken from at least 3 cm outside the tumour edge. All specimens were acquired directly after excision and dissection from the patient, and were immediately stabilised by snap-freezing in cryovials and immersion in liquid nitrogen. Samples were stored at −80 °C until analysis. For western blot analysis, we randomly selected two surgical CRC samples from patients with no preoperative chemotherapy or irradiation among the samples collected at Osaka University Hospital in 2016. All patients provided written informed consent, and this study was approved by the Ethics Board of Osaka University Hospital. The standard protocols for patient follow-up included blood tests every 3 months, computerised tomography every 6 months and colonoscopy every other year, with follow-up continuing until 5 years after surgery. Tumour stages were defined according to the tumour node metastasis staging system, 7th edition.^23^

### Immunohistochemical staining

Immunohistochemical staining was performed as previously described.^24^ We assessed syntenin-1 protein expression by immunohistochemical staining of formalin‑fixed and paraffin‑embedded normal colorectal mucosa and colorectal cancer tissue sections. The surgical tissue samples were incubated overnight at room temperature in 10% formalin, and then embedded in paraffin. Samples were cut into 3.5-µm-thick tissue sections, and endogenous peroxidase activity was blocked using the VECTASTAIN Elite ABC Horseradish Peroxidase Kit (Rabbit IgG; #PK‑6101; Vector Laboratories, Burlingame, CA, USA), with a 20 min incubation at room temperature. Then, the tissue slices were incubated overnight at 4 °C with rabbit polyclonal anti‑syntenin antibody (dilution, 1:1000; #ab133267; Abcam, Cambridge, UK). Haematoxylin was used for nuclear staining for 1 min. Dehydration was performed using 60, 70, 80, 90 and 95% ethanol for 1 min each; 100% ethanol for 2 min, twice; and xylene for 5 min, three times. The specimens were visualised on the light field using a confocal microscope BZ‑X710 (Keyence Corporation, Osaka, Japan) and a BZ‑X analyser (v. 1.3.0.3; Keyence Corporation). We manually counted the number of syntenin-1-positive cells among the epithelial cancer cells in three randomly selected areas at ×40 magnification. The cut-off point for positive was set as a value more than the average number of positive cells in all tumour samples, and more than the number of positive cells in adjacent tissues.

### Cell lines and cell culture

We obtained the human embryonic kidney epithelial cell line HEK-293T, and the CMS4-classified human CRC cell lines SW480, SW620 and Caco2^25^ from the Biochemistry and Molecular Biology Laboratory of Yunnan University, P.R. China. These cells were cultured in Dulbecco’s modified Eagle’s medium (DMEM; Sigma-Aldrich; Merck KGaA, Darmstadt, Germany) containing 10% foetal bovine serum (FBS; Gibco; Thermo Fisher Scientific, Waltham, MA, USA) at 37 °C in a humidified incubator containing 5% CO_2_.

### RNA interference

Syntenin-1 short hairpn RNA (shRNA) (sh1: TRCN0000029154; sh2: TRCN0000029155; Sigma) and a control scrambled shRNA (Sigma) were inserted into the pLKO-tetpuro vector (Plasmid #21915; Addgene, Cambridge, MA, USA), and transfected into 293FT cells with packaging plasmids using Lipofectamine 3000 (Thermo Fisher Scientific), following the manufacturer’s protocol. At 48 h after transfection, the supernatant was filtered and used for virus transduction to SW480, SW620 and Caco2 cells with 5 µM polybrene (Sigma-Aldrich). Cells transduced with the vectors were selected for growth in 1 µg/mL puromycin. We also used PTGER2-specific small interfering RNA (siRNA; Sigma-Aldrich) to knockdown PTGER2 messenger RNA. PTGER2, or the negative control siRNA, was transfected into SW480, SW620 and Caco2 cells at a final concentration of 50 nM with Lipofectamine RNAiMax (Thermo Fisher Scientific) following the manufacturer’s protocol. RNA was extracted at 48 h after transfection.

### Quantitative real-time reverse transcriptase-polymerase chain reaction

We extracted total RNA from cultured cells using TRIzol® RNA Isolation Reagents (Thermo Fisher Scientific) as previously described.^26^ From 10 ng total RNA, we synthesised complementary DNA (cDNA) using a High Capacity RNA-to-cDNA Kit (Thermo Fisher Scientific) following the manufacturer’s protocol. Polymerase chain reaction (PCR) was performed in a Light CyclerTM 2.0 System (Roche Applied Science) using the Thunderbird® SYBR® quantitative PCR mix (Toyobo Life Science, Osaka, Japan). Data from each experiment were normalised to the expression of a control gene (*ACTB*). The following primers were used: SDCBP, forward 5′-TGGTGGCTCCTGTAACTGGTAA-3′ and reverse 5′-TGCATGGTAATCGTCCGTTCAA-3′; PTGER2, forward 5′-GTCTGCTCCTTGCCTTTCAC-3′ and reverse 5′-CCTCAAAGGTCAGCCTGTTT-3′; and β-actin, forward 5′-GATGAGATTGGCATGGCTTT-3′ and reverse 5′-CACCTTCACC GTTCCAGTTT-3′.

### Western blot analysis

The Western blot assay was performed as previously described.^27^ From the cells and clinical samples, we extracted proteins in radio-immunoprecipitation assay (RIPA) buffer (Thermo Fisher Scientific, Waltham, MA, USA). Clinical samples were homogenised in RIPA buffer using an ultrasonic disruptor (UD-201; Tomy Seiko, Co., Ltd, Tokyo, Japan). Proteins were separated by electrophoresis on sodium dodecyl sulfate-polyacrylamide gel electrophoresis Tris-HCl gels (Bio-Rad Laboratories, Hercules, CA, USA), and then transferred onto polyvinylidene difluoride membranes and blocked with 5% skim milk in Tris-buffered saline with Tween-20 at room temperature for 1 h. The blots were incubated for 2 h with primary antibodies, and then for 1 h at room temperature with horseradish peroxidase-linked anti-rabbit or anti-mouse IgG (dilution, 1:100,000; GE Healthcare Biosciences, Piscataway, NJ, USA). The antigen–antibody complex was detected using the ECL Prime Western Blotting Detection Kit (GE Healthcare Biosciences). Western blot analyses were performed using anti-syntenin-1 (#ab133267; Abcam, Cambridge, UK) and anti-β-actin (A2066; Sigma-Aldrich, St. Louis, MO, USA) antibodies.

### RNA-seq and bioinformatics analysis

From SW480 cells transfected with shRNA duplex specific for SDCBP or non-targeting shRNA, total RNA was extracted using an RNeasy Mini Kit (Qiagen, Hilden, Germany) following the manufacturer’s instructions. Sequencing was performed on an Illumina HiSeq 2500 platform in 75-base single-end mode. Microarray data were analysed using QIAGEN’s Ingenuity Pathway Analysis (IPA) software (Qiagen, Redwood City, USA). The data were normalised by the percentile shift to the 75th percentile, and the threshold raw signal was set to 1.0. Data were filtered using a fold-change cut-off of 2.0 and *p* ≤ 0.05. The scores associated with each form of analysis were calculated using the logarithm of the *p* value (Fisher’s exact test). A *p* value of <0.05 was set as significant.

### Proliferation assay

The proliferation assay was performed as previously described.^28^ Cells were seeded at a density of 4 × 10^3^ cells per well in 96-well plates. From 24 to 96 h after seeding, cell proliferation was assessed at 24-h intervals using Cell Counting Kit-8 (Dojindo Molecular Technologies, Inc., Kumamoto, Japan) following the manufacturer’s protocol.

### Scratch wound healing assay

The scratch wound healing assay was performed as previously described.^29^ Cells were seeded at a density of 5 × 10^5^ cells/well in 6-well plates and grown to confluence under standard conditions. To perform the scratch assay, a 200-μL pipette tip was run through the dish, and then the cells were cultured under standard conditions, except that DMEM with 1% FBS was used to prevent proliferation. Prior to photographing the plates, they were washed with fresh DMEM with 1% FBS to remove non-adherent cells. Cell migration was evaluated by measuring the average distances between the wound edges at 10 random areas.

### Sphere formation assay

The sphere formation assay was performed as previously described.^27^ We evaluated the ability of cells to form spheres in Dulbecco’s modified Eagle’s medium/Nutrient Mixture F-12 (DMEM/F12) supplemented with 20 ng/mL epidermal growth factor (Invitrogen), 20 ng/mL human platelet growth factor (Sigma-Aldrich) and 1% antibiotic–antimycotic solution (Invitrogen). Single cells were plated at a concentration of 200 cells/well (shRNA) or 1000 cells/well (siRNA) in each well of a 96-well ultralow attachment plate (Corning Life Sciences, Acton, MA, USA), and cultured in a 37 °C incubator supplied with 5% CO_2_. On the 20th day (shRNA) or 6th day (siRNA), we evaluated sphere-forming ability by counting the number of spheres of ≥50 µm in each well.

### Chemosensitivity assay

The chemosensitivity assay was performed as previously described.^28^ Cells were seeded at a density of 4 × 10^3^ cells/well in 96-well plates, and precultured for 24 h. Next, the cells were exposed to various concentrations of 5-fluorouracil (5-FU; Tokyo Chemical Industry Co., Ltd, Tokyo, Japan) and oxaliplatin (L-OHP; Yakult Honsha Co., Ltd, Tokyo, Japan) for 72 h at 37 °C in a humidified incubator containing 5% CO_2_. We evaluated the in vitro cytotoxic effects of 5-FU and L-OHP using the Cell Counting Kit-8 (Dojindo Molecular Technologies, Inc., Kumamoto, Japan), following the manufacturer’s protocol. The viability data of CRC cells treated with each concentration of L-OHP were used to calculate the half-maximal inhibitory concentration values.

### Statistical analysis

All evaluation data were presented as the mean ± SEM obtained from at least three independent experiments. We evaluated the statistical differences among different assay groups using the two-tailed unpaired Student’s *t* test. Statistical calculations were performed using JMP® Pro 13.1.0 software (SPSS, Inc., Chicago, IL, USA). A *p* value of <0.05 was considered to indicate statistical significance.

### Statement in the methods

This study was approved by the Ethics Board of Osaka University Hospital (No. 15218-4). All methods were performed in accordance with the relevant guidelines and regulations.

## Results

### Higher syntenin-1 expression in tumour tissues than non-tumour tissues in CRC

**Fig. 1:**
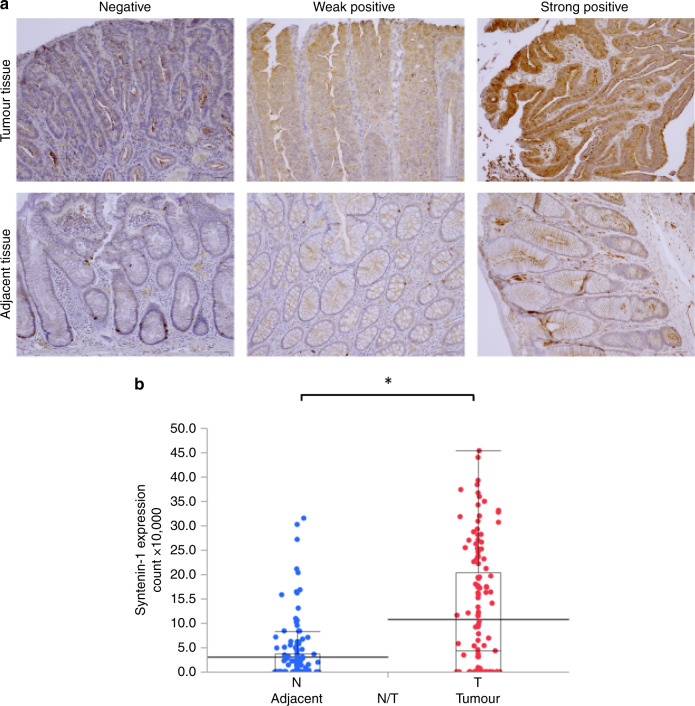
Syntenin-1 expression is associated with cancer. **a** Immunohistologic staining was performed to detect syntenin-1 expression in tumour and adjacent non-tumour tissues of the human colon. Data represent the quantitative analyses of paired clinical samples (scale bar, 50 μm). **b** Quantitative graphs indicate plots of the number of positive cells for each case. Statistical significance was determined by Student’s *t* test; *p < 0.05.

Using human CRC tumour tissue microarrays, we found that syntenin-1 expression was higher in tumour tissues than in adjacent non-tumour tissues (Fig. 1a, b). Western blot analysis of two randomly selected CRC surgical samples revealed that syntenin-1 expression was higher in tumour tissue than in normal colon mucosa (Supplementary Fig. 1).

### Correlation of syntenin-1 expression with sphere formation, L-OHP chemoresistance and migratory ability

**Fig. 2:**
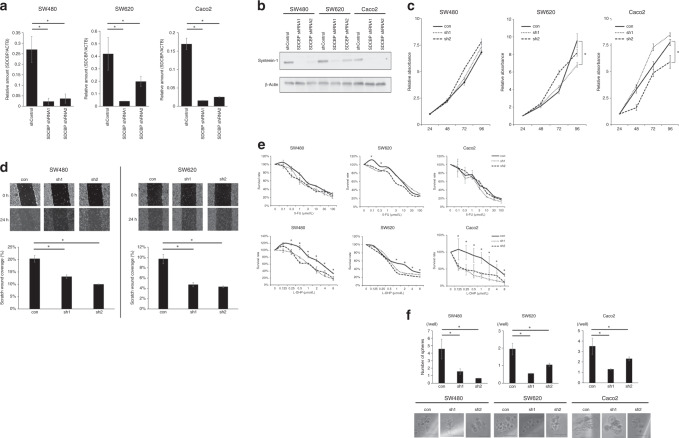
Syntenin-1 is associated with the malignant potential of cancer. **a** Syntenin-1 expression levels in SW480, SW620 and Caco2 cells transfected with negative control shRNA or shRNAs against SDCBP. Reverse transcription-quantitative polymerase chain reaction was performed to determine mRNA expression levels, which were normalised to ACTB. Results represent the mean ± SD of three independent experiments. *p < 0.05. **b** Western **b**lotting of syntenin-1 in cell lines transfected with negative control shRNA or shRNAs against SDCBP. **c** Proliferation of cells transfected with shRNA targeting SDCBP expression. Results represent the mean ± SD of three independent experiments. **d** Representative images of the scratch wound healing assay in SW480 and SW620 cells transfected with negative control shRNA or shRNAs against syntenin-1 (upper panel; magnification, ×100). Distances between wound edges at the indicated time-points (normalised to distance at 0 h) are shown as the average of eight different areas (lower panel). *p < 0.05 vs. negative control. **e** Chemosensitivity assay of cells transfected with shRNA targeting SDCBP expression. Chemosensitivity was indicated by viability after treatment with L-OHP or 5-FU for 72 h. Results represent the mean ± SD of three independent experiments. *p < 0.05. **f** Sphere formation by cells transfected with shRNA targeting SDCBP expression. Lower panel, representative images of a sphere (scale bar, 50 µm). *p < 0.05. con negative control, sh1 and sh2 short hairpin1 and 2 against syntenin-1, L-OHP oxaliplatin, 5-FU fluorouracil.

To elucidate how syntenin-1 contributes to the malignant potential of CRC, we performed in vitro loss-of-function assays using RNA interference. PCR and western blot analysis demonstrated high transfer efficiency of the shRNA targeting syntenin-1 (Fig. 2a, b). In the CRC-derived cell lines SW480, SW620 and Caco2, syntenin-1 knockdown significantly inhibited migratory ability (Fig. 2d), but did not affect proliferation rate (Fig. 2c). Syntenin-1 downregulation significantly reduced L-OHP chemoresistance in all three cell lines compared to controls, whereas chemosensitivity to 5-FU was not affected (Fig. 2e). The sphere formation assay revealed a reduced presence of CSCs among syntenin-1-knockdown cells compared to in controls (Fig. 2f).

### PTGER2 expression was decreased in syntenin-1-knockdown cells

**Fig. 3:**
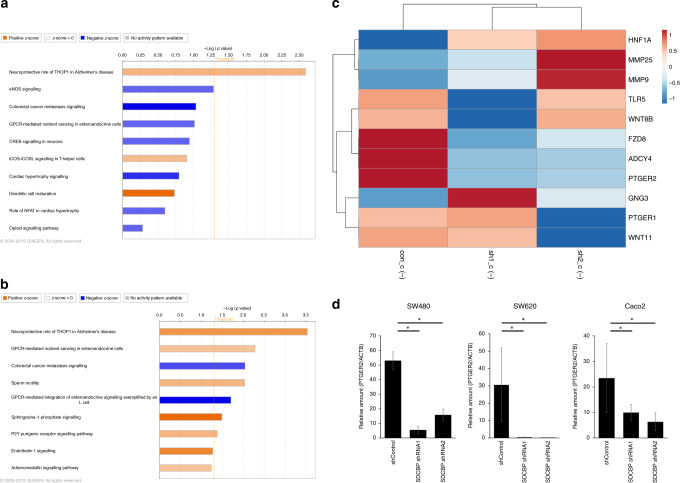
Syntenin-1 expression is associated with prostaglandin E2 receptor 2 (EP2) expression. **a**, **b** Ingenuity pathway analysis (IPA) revealed the top canonical pathways of SW480 with silenced syntenin-1 expression—with shRNA1 (**a**) or shRNA2 (**b**)—compared to negative control SW480. **c** Heatmap of the genes that were differentially expressed in syntenin-1-silenced SW480, within the canonical pathways of colorectal cancer metastasis signalling that were identified in the IPA analysis. **d** PTGER2 mRNA expression in syntenin-1-knockdown cells. Results represent the mean ± SD of three independent experiments. *p < 0.05.

We performed microarray analysis to identify genes related to syntenin-1. We used SW480 to downregulate syntenin-1 expression, and analysed the microarray results using IPA to identify the predominant canonical pathways. Figure 3a, b shows the molecular and cellular functions and canonical pathways with high absolute *Z*-score values. We focused on the colorectal cancer metastasis signalling pathway, which had a high *Z*-score with both shRNAs. Mapping the genes in the colorectal cancer metastasis signalling pathway revealed that syntenin-1 downregulation led to downregulation of the membrane proteins Frizzled and PTGER2 (Fig. 3c and Supplementary Fig. 2a, b). PCR revealed that PTGER2 expression was inhibited by syntenin-1 downregulation in all three cell lines (Fig. 3d).

### Silencing PTGER2 reduced presence of CSCs and L-OHP chemoresistance

**Fig. 4:**
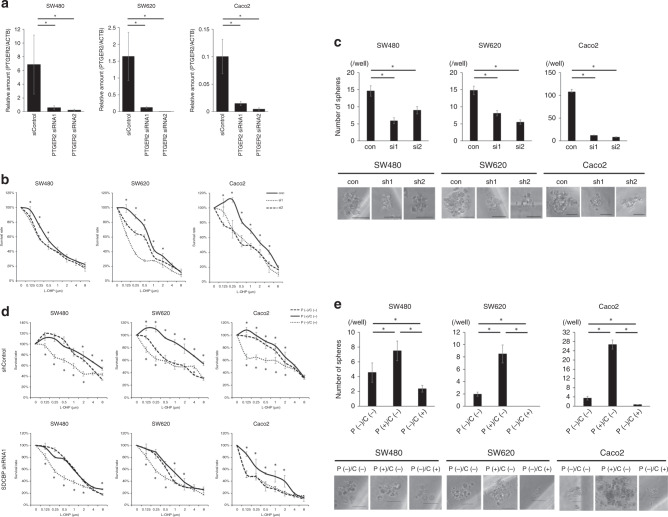
Coexistence of EP2 and syntenin-1 is associated with L-OHP chemosensitivity and sphere formation of cancer. **a** PTGER2 expression levels in SW480, SW620 and Caco2 cells transfected with negative control siRNA or siRNAs against PTGER2. Reverse transcription-quantitative polymerase chain reaction was performed to detect mRNA expression levels, which were normalised to ACTB. Results represent the mean ± SD of three independent experiments. *p < 0.05. **b** Chemosensitivity assay of cells transfected with siRNA targeting PTGER2 expression. Chemosensitivity was indicated by viability after treatment with L-OHP for 72 h. Results represent the mean ± SD of three independent experiments. *p < 0.05. **c** Sphere formation by cells transfected with siRNA targeting PTGER2 expression. Lower panel, representative images of a sphere. Scale bar, 50 µm. *p < 0.05. **d** Chemosensitivity assay of cells transfected with shRNA targeting SDCBP expression with/without prostaglandin E2 or celecoxib. Chemosensitivity was indicated by viability after treatment with L-OHP for 72 hours. Results represent the mean ± SD of three independent experiments. *p < 0.05. **e** Sphere formation by cells transfected with shRNA targeting SDCBP expression with/without prostaglandin E2 or celecoxib. Lower panel, representative images of a sphere. Scale bar, 50 µm. *p < 0.05.

To confirm the involvement of PTGER2 in the syntenin-1 knockdown-mediated decreases of CSCs and chemoresistance, we conducted in vitro functional assays using siRNA-mediated PTGER2 knockdown. We first transfected SW480, SW620 and Caco2 cells with two individual siRNA duplexes specific for PTGER2 (siPTGER2 #1, #2), which yielded high transfer efficiency (Fig. 4a). Next, we compared L-OHP chemosensitivity between the PTGER2-knockdown cells and control cells. In all SW480, SW620 and Caco2 cells, PTGER2 knockdown resulted in decreased chemoresistance to L-OHP (Fig. 4b). We next performed a sphere formation assay to evaluate the presence of CSCs, and found that PTGER2 knockdown resulted in reduced presence of CSCs (Fig. 4c). These results indicated that PTGER2 knockdown in SW480, SW620 and Caco2 cells induced the same phenotype as syntenin-1 knockdown, suggesting that the decreased CSCs and chemoresistance observed with syntenin-1 knockdown was caused by reduced expression of PTGER2.

### Syntenin-1 expression and reaction of PTGER2 with prostaglandin is associated with presence of CSCs and L-OHP chemoresistance

PTGER2 exerts effects by binding to PGE2.^9^ We postulated that syntenin-1 expression, and the associated PTGER2 expression, caused a change in responsiveness to PGE2 among colorectal cancer cells. To examine this hypothesis, we incubated syntenin-1-knockdown cells and control cells with PGE2 or celecoxib, which exerts anti-inflammatory effects by targeting COX-2. The results indicated that PGE2-treated control cells exhibited reduced L-OHP chemosensitivity, while celecoxib-treated SW480, SW620 and Caco2 cells showed increased L-OHP chemosensitivity (Fig. 4d). Additionally, the presence of CSCs was reduced by celecoxib treatment, and increased by PGE2 treatment (Fig. 4e). Compared to control cells, syntenin-1-knockdown cells showed reduced reactivity to PGE2 or celecoxib in terms of L-OHP chemosensitivity (Fig. 4d).

### Clinical significance of syntenin-1 in CRC

**Fig. 5:**
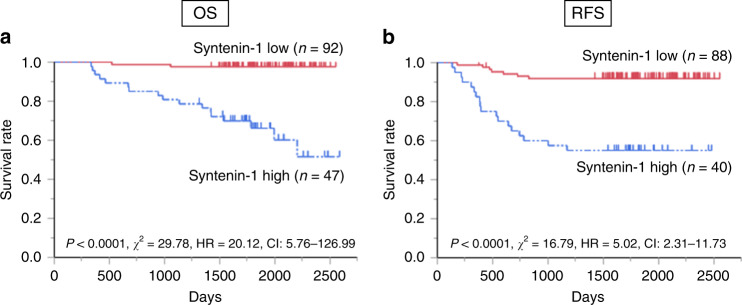
Kaplan–Meier curves for OS or RFS according to syntenin-1 expression. **a** Cumulative OS for all cases. **b** Cumulative RFS for cases of curative resection. High and low syntenin-1 expression groups were separated based on the immunohistologic staining score of syntenin-1 expression. OS overall survival, RFS recurrence‑free survival.

Using samples from 139 CRC patients, we analysed the relationships between syntenin-1 expression and clinicopathological features (Table 1). The patients were divided into two groups based on the average number of syntenin-1-positive cells/fields in the tumour samples. Compared to the low expression group, the high syntenin-1 expression group included more patients with less differentiated histologic grade (*p* = 0.001). The groups did not significantly differ in any other clinicopathological factors. There were no cases involving preoperative treatment for rectal cancer. With regards to postoperative chemotherapy, 5-FU was administered in 26 cases in the syntenin-1 high group and 35 cases in the syntenin-1 low group, and L-OHP was administered in 6 cases in the syntenin-1 high group and 3 cases in the syntenin-1 low group. Compared to the high expression group, the low expression group had a significantly longer overall survival (OS) (*p* < 0.0001) and recurrence‑free survival (RFS) (*p* < 0.0001; Fig. 5). Univariate and multivariate analysis showed that syntenin-1 expression was a significant prognostic factor for both OS (*p* < 0.0001; Table 2) and RFS (*p* < 0.0001; Table 3).

**Table 1:** Baseline characteristics of the syntenin-1 high and low groups.

Clinicopathological variables	Syntenin-1 expression		*P* value
	Low expression (*n* = 92)	High expression (*n* = 47)	
Gender; male/female, *n*	53/39	29/18	0.64
Age (year), mean (SD)	65.5 ± 10.4	62.94 ± 10.9	0.18
CEA (ng/mL), median (range)	3 (1–527)	4 (1–2841)	0.86
CA19-9 (U/mL), median (range)	13 (3–12777)	17 (0–1661)	0.55
Tumour size (mm), median (range)	38 (8–120)	43 (16–100)	0.50
Location, *n*			
Colon/rectum	56/36	26/11	0.53
Histological grade, *n*			
Tub1, tub2, pap/por, muc	90/2	38/9	0.001
T grade, *n*			
T0, 1, 2/T3, 4	41/51	15/32	0.20
Lymphatic invasion, *n*			
Absent/present	28/64	10/37	0.25
Venous invasion, *n*			
Absent/present	70/22	34/13	0.68
Lymphatic metastasis, *n*			
Absent/present	50/42	21/26	0.28
Liver metastasis, *n*			
Absent/present	86/6	39/8	0.07
Peritoneum dissemination, *n*			
Absent/present	91/1	45/2	0.26
Lung metastasis, *n*			
Absent/present	90/2	45/2	0.55
Other organ metastasis, *n*			
Absent/present	92/0	45/2	0.11
Stage, *n*			
0, I, II/III, IV	49/43	17/30	0.056

*CEA* carcinoembryonic antigen, *CA* cancer antigen.

**Table 2:** Results of univariate and multivariate analyses for overall survival in a Cox proportional hazards model.

Clinicopathological variables	Univariates analysis			Multivariate analysis		
	Hazard ratio	95% CI	*P* value	Hazard ratio	95% CI	*P* value
Age (years)						
>64/≤64	0.69	0.27–1.70	0.42			
Gender						
Female/male	1.03	0.40–2.55	0.95			
CEA (ng/mL)						
>3/≤3	3.91	1.50–12.1	0.048	2.03	0.73–6.65	0.18
CA19-9 (U/mL)						
>14/≤14	6.89	2.29–29.6	0.0003	3.92	1.11–18.7	0.0323
Tumour size (mm)						
>40 vs ≤40	1.32	0.53–3.32	0.546			
Location						
Rectum/colon	1.27	0.50–3.16	0.6			
Histological grade						
Por muc/tub1, tub2, pap	5.72	1.80–15.8	0.005	1.65	0.50–4.77	0.385
T grade						
T3, 4/T0, 1, 2	6.4	1.83–40.3	0.0019	1.81	0.43–12.6	0.448
Lymphatic invasion						
Positive/negative	3.79	1.08–23.9	0.0352	1.68	0.30–13.3	0.569
Venous invasion						
Positive/negative	1.99	0.74–4.96	0.166			
Lymphatic metastasis						
Positive/negative	3.06	1.17–9.48	0.0218	1.7	0.54–6.98	0.383
Syntenin-1 expression						
High/low	19.8	5.65–124.7	<0.0001	16.2	4.48–103.7	<0.0001

*95% CI* 95% confidence interval, *CEA* carcinoembryonic antigen, *CA* cancer antigen.

**Table 3:** Results of univariate and multivariate analyses for recurrence-free survival in a Cox proportional hazards model.

Clinicopathological variables	Univariates analysis			Multivariate analysis		
	Hazard ratio	95% CI	*P* value	Hazard ratio	95% CI	*P* value
Age						
>64/≤64	0.6	0.26–1.32	0.2			
Gender						
Female/male	0.75	0.34–1.67	0.48			
CEA (ng/mL)						
>3/≤3	2.2	1.01–5.11	0.047	1.71	0.76–3.98	0.19
CA19-9 (U/mL)						
>14/≤14	1.31	0.591–2.89	0.495			
Tumour size (mm)						
>40 vs ≤40	1.69	0.77–3.81	0.188			
Location						
Rectum/colon	1.1	0.49–2.43	0.79			
Histological grade						
Por muc/tub1, tub2, pap	1.99	0.47–5.77	0.3			
T grade						
T3, 4/T0, 1, 2	6.53	2.26–27.6	0.0002	3.19	0.97–14.8	0.057
Lymphatic invasion						
Positive/negative	5.33	1.57–33.2	0.0042	0.97	0.20–7.16	0.98
Venous invasion						
Positive/negative	3.81	1.71–8.42	0.0014	2.93	1.24–7.09	0.0146
Lymphatic metastasis						
Positive/negative	5.19	2.1–15.6	0.0002	2.61	0.88–9.76	0.084
Syntenin-1 expression						
High/low	7.08	3.0–18.2	<0.0001	8.47	3.6–22.3	<0.0001

*95% CI* 95% confidence interval, *CEA* carcinoembryonic antigen, *CA* cancer antigen.

## Discussion

Increased syntenin-1 expression has been detected in several malignancies, including melanoma, breast cancer and glioma;^30–34^ however, the common mechanism through which increased syntenin-1 expression affects the cancerous properties of cells remains unclear. In the present study, we determined the importance of syntenin-1 and identified a novel association between syntenin-1 and tumour cell properties in colorectal cancer. Notably, we found that syntenin-1 promoted tumour cell chemosensitivity to L-OHP, presence of CSCs and migration through the regulation of PTGER2 expression. Moreover, celecoxib suppressed L-OHP chemosensitivity and CSCs in cells with higher syntenin-1 expression, suggesting that syntenin-1 regulated the reaction between PTGER2 and prostaglandin.

Our present results also demonstrated that higher syntenin-1 expression was significantly correlated with poorer OS and RFS. To our knowledge, this study is the first to clarify the correlation between syntenin-1 expression and prognosis in colorectal cancer, and to demonstrate that syntenin-1 may be a marker for poor prognosis in this disease. The correlation between syntenin-1 expression and prognosis has been previously reported in several other cancers.^30,31,35^ In breast cancer and urothelial cancer, high syntenin-1 expression is associated with more lung metastases.^12,30^ However, our present results showed no syntenin-1-associated difference in metastasis. Moreover, our examination results indicated that lymph node involvement was not an independent prognostic factor for OS or RFS. It is considered that the lymph node metastasis was not significant in the multivariate analysis, because the cases without lymph node metastasis had a small number of deaths (in the high expression group, N0 had 17 survivors (80.95%) and 4 deaths (19.05%), while in the low expression group, N0 had 49 survived (98.00%) and 1 died (2.00%)). This is clinically natural, and we do not consider the study population to be a unique population. On the other hand, there is a quantitative interaction between lymph node metastasis and syntenin-1 expression (in the high expression group, N1 had 13 survivors (50.00%) and 13 deaths (50.00%), while in the low expression group, N1 survived 41 (97.62%) and died 1 (2.38%)), indicating that the high expression group of patients with lymph node metastasis has a strong poor prognosis. It may be possible to evaluate the effect of lymph node metastasis by increasing the number of cases. Additionally, we found that venous invasion was not an independent prognostic factor for OS. However, there were few cases of venous invasion overall, which may have influenced the lack of significant relationship with OS. Although syntenin-1 expression has been reported to increase cancer proliferation, migration and invasion in some cancer cell lines,^12,30,33–35^ its malignant potential and its effects on chemoresistance and tumorigenicity have not been sufficiently explored. Syntenin-1 is related to cancer migration, tumorigenic and chemosensitivity, but not to proliferation—which is likely an indication that syntenin-1 functions in epithelial-to-mesenchymal transition (EMT) or autophagy. However, suppressing syntenin-1 expression did not lead to different expression of EMT or autophagy-related molecules (data not shown). To examine how syntenin-1 affected tumour cell properties in colorectal cancer, we conducted microarray analysis by IPA using SW480 with downregulated syntenin-1, and we found that PTGER2 was suppressed in conjunction with syntenin-1 (Fig. 3c and Supplementary Fig. 2a, b).

PTGER2 belongs to a family of seven transmembrane G protein-coupled receptors and exerts effects by binding to PGE2. PGE2 reportedly promotes tumour epithelial cell proliferation, survival and migration/invasion via multiple signalling pathways.^6–8^ Wang and co-workers^9^ suggested that PGE2 promotes CRC initiation, growth and metastasis via CSCs, and that PTGER4 mediates the effects of PGE2 on the induction of colonic CSC expansion and promotion of liver metastasis. Another study demonstrated that PGE2 release induced by chemotherapy promoted neighbouring CSC repopulation, and that this repopulation could be abrogated by celecoxib-mediated blockade of PGE2 signalling.^36^ The most meaningful finding in our present study was that under syntenin-1-expressing conditions, CRC cell lines showed changes in L-OHP chemosensitivity and CSCs upon loading with prostaglandins or suppression by celecoxib. In contrast, such changes were suppressed when syntenin-1 expression was suppressed. These findings indicated that, in CRC with high syntenin-1 expression (which showed a poor prognosis), the inflammatory state increases CRC malignancy, and celecoxib suppresses CRC malignancy.

The present study had several limitations. First, since we lacked a good antibody for PTGER2, we could not evaluate PTGER2 protein expression by western blot analysis. Second, data were not available regarding the Kras/Braf mutational status or MSI status of our clinical samples; thus, we could not examine their effects. Third, our results did not identify a direct or indirect connection between syntenin-1 and PTGER2. Syntenin-1 is a small protein containing two closely linked PDZ domains, which interact by directly binding to membrane proteins—for example, with syndecan or certain Frizzled receptors.^37^ Thus, we hypothesise that syntenin-1 and PTGER2 bind either directly or through a mediator molecule. Further exploration is required to determine the direct involvement of syntenin-1 and PTGER2 in CRC.

## Conclusions

In conclusion, our present results demonstrated that syntenin-1 is related to the CSC properties of colon cancer cells through regulation of PTGER2. Moreover, we suggest that celecoxib is a promising candidate for treating CRC with high syntenin-1 expression, which has a poor prognosis.

## Supplementary information

Supplemental figure 1 & 2

## Data Availability

The data supporting the findings of this study are available from the corresponding author upon reasonable request.
