# Supplementary material for: Syntenin-1 promotes colorectal cancer stem cell expansion and chemoresistance by regulating prostaglandin E2 receptor
Source: Br J Cancer. 2020 Jun 29;123(6):955–64. doi: 10.1038/s41416-020-0965-9 (PMC7492211; doi:10.1038/s41416-020-0965-9)
Supplement: Supplementary file 1 — Supplemental figure 1 & 2 [file 41416_2020_965_MOESM1_ESM.pptx]

## Slide 1
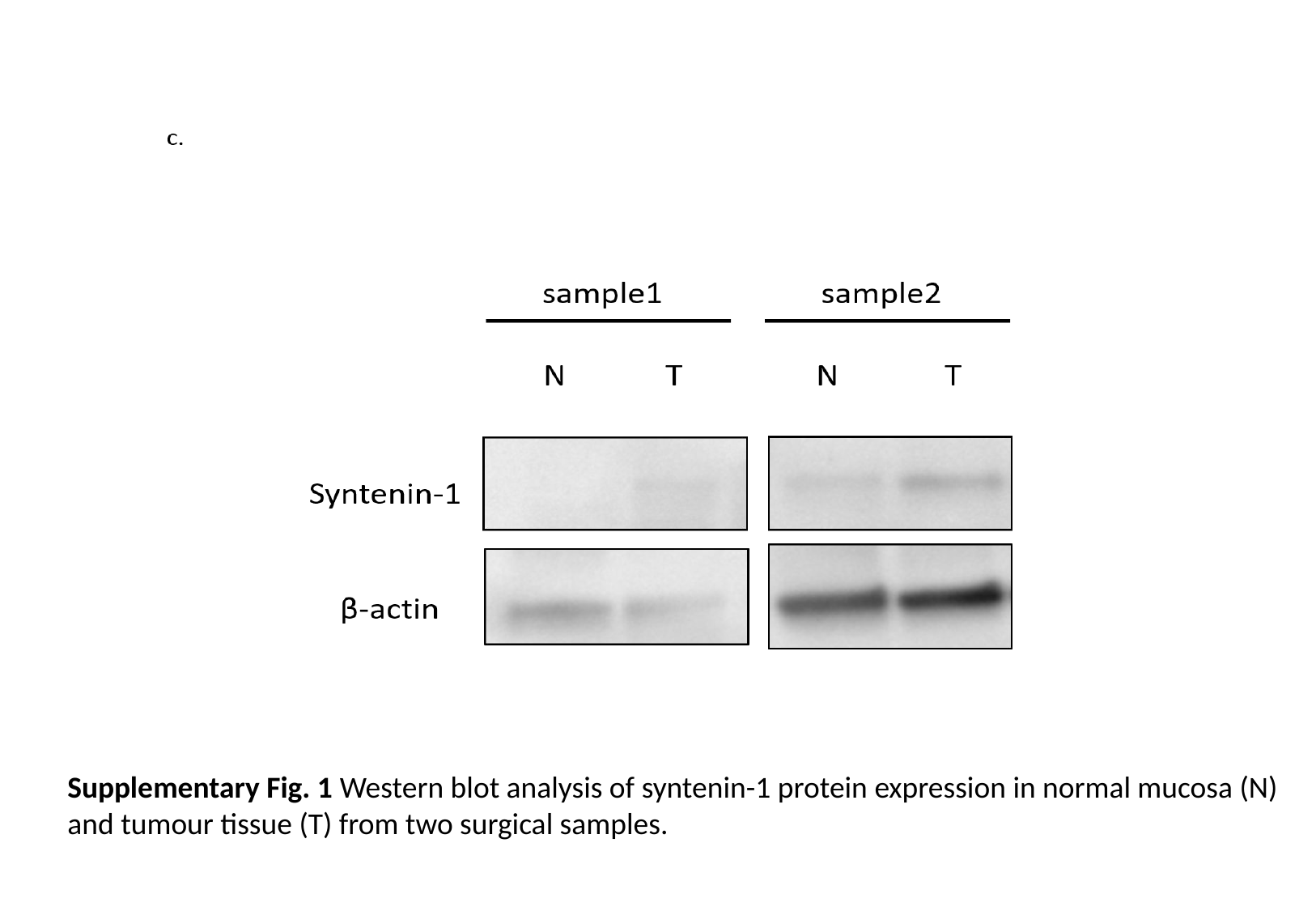

Supplementary Fig. 1 Western blot analysis of syntenin-1 protein expression in normal mucosa (N)
and tumour tissue (T) from two surgical samples.

## Slide 2
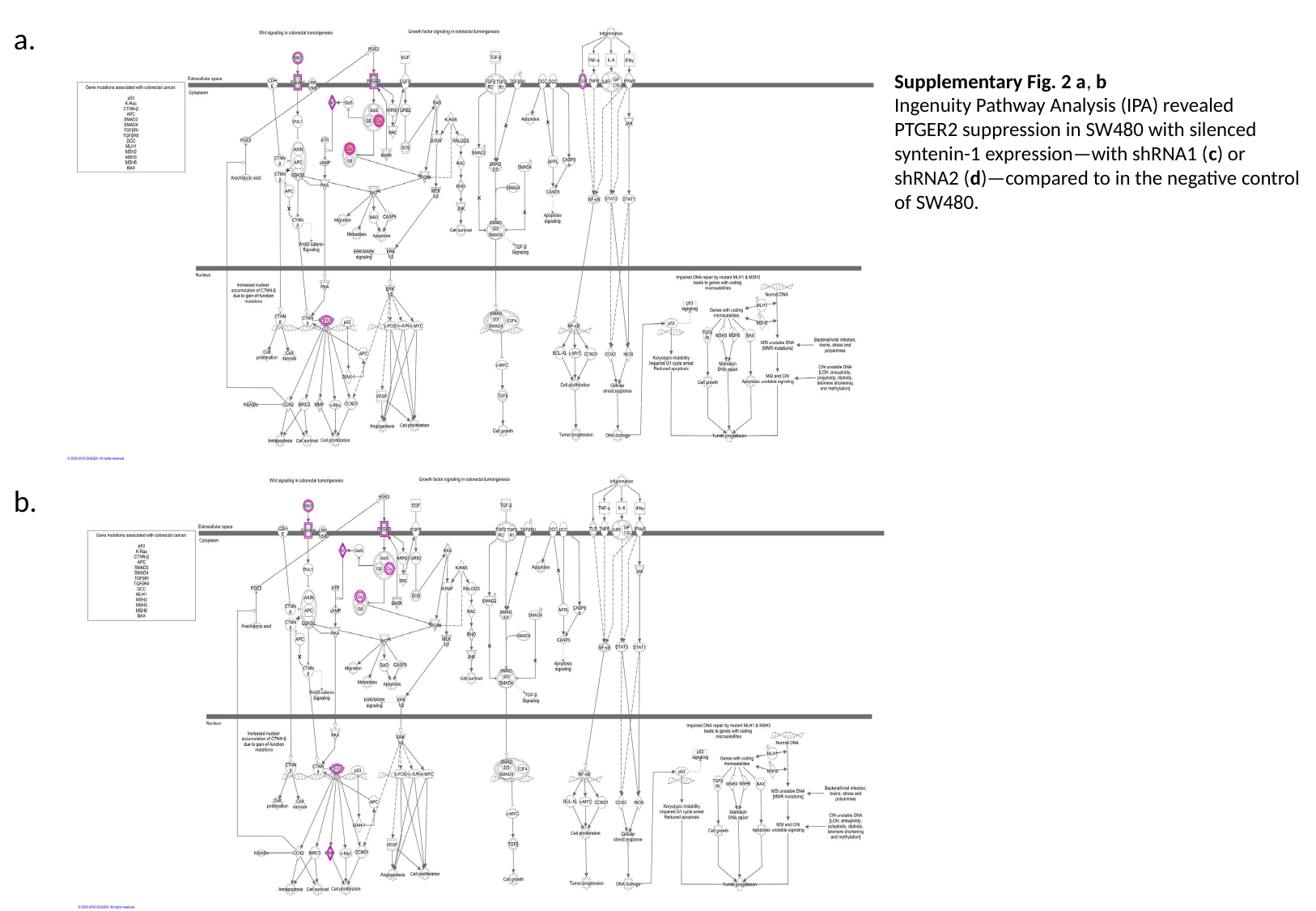

a.
Supplementary Fig. 2 a, b
Ingenuity Pathway Analysis (IPA) revealed
PTGER2 suppression in SW480 with silenced
syntenin-1 expression—with shRNA1 (c) or
shRNA2 (d)—compared to in the negative control
of SW480.
b.
